# Integrative Mutational Landscape of Mycosis Fungoides Using a National Genomics Repository

**DOI:** 10.3390/cancers17182984

**Published:** 2025-09-12

**Authors:** Grace S. Saglimbeni, Beau Hsia, Peter T. Silberstein, Abubakar Tauseef

**Affiliations:** 1 Department of Hematology and Oncology, Creighton University School of Medicine, Phoenix, AZ 85012, USA; gracesaglimbeni@creighton.edu (G.S.S.); beauhsia@creighton.edu (B.H.); 2 Division of Hematology and Oncology, Department of Internal Medicine, Creighton University Medical Center, Omaha, NE 68124, USA; petersilberstein@creighton.edu

**Keywords:** mycosis fungoides, AACR Project GENIE, somatic mutations, *FAT1*, *JAK*, p53, epigenetic regulators, biomarker discovery, cancer genomics

## Abstract

Background: Mycosis fungoides (MF) is a rare cutaneous T-cell lymphoma (CTCL) that presents clinically on the skin as patches, plaques, or tumors. MF often mimics benign inflammatory conditions which leads to difficult and delayed diagnosis, worsening prognosis despite available treatment options. This study seeks to improve diagnosis and identify potential therapeutic targets by better characterizing MF’s genetic landscape using the AACR Project GENIE dataset. Methods: Retrospective analysis of MF cases was conducted using the AACR Project GENIE database accessed from cBioPortal (v17.0-public) on 5 June 2025. Data analysis included identifying recurrent somatic mutations, assessing patterns of mutation co-occurrence and mutual exclusivity using non-parametric tests with Benjamini–Hochberg False Discovery Rate (FDR) correction, and examining enrichment of specific mutations based on sex and race, with significance of *p* < 0.05. Results: Recurrent alterations included *FAT1* (28.2%), *KMT2D* (19.2%), *TP53* (13.5%), *JAK3* (11.5%), and *SETBP1* (11.5%), highlighting the role of Wnt signaling, epigenetic dysregulation, the p53 pathway, and JAK/STAT signaling in MF pathogenesis. Mutations with significant co-occurrence and enrichment in White, Black, and Asian populations were identified. Conclusions: The findings of this study provide a comprehensive understanding of MF’s molecular profile. The discovery of commonly mutated pathways (Wnt, p53, JAK/STAT, and epigenetic regulators) suggests potential targets for the development of future therapies. Furthermore, the enrichment of certain mutations based on race and patterns of alteration co-occurrence offer possibilities for patient-tailored treatment approaches.

## Simple Summary

Mycosis fungoides (MF) is a primary T-cell lymphoma that manifests on the skin. Using the American Association for Cancer Research (AACR) Project Genomics, Evidence, Neoplasia, Information, Exchange (GENIE), a publicly accessible genomic repository, this study explores the mutational profile of MF in a large, diverse patient cohort. This research uncovers the most common somatic mutations in *FAT1*, *KMT2D*, *TP53*, *JAK3*, and *SETBP1*, which disrupt key pathways controlling cell adhesion, chromatin structure, p53 activity, and cytokine signaling. These alterations provide insight into disease mechanisms and reveal potential diagnostic, prognostic, and therapeutic targets. Significant co-mutation patterns suggest cooperation between signaling and epigenetic pathways, while the identification of mutation enrichments across racial groups provides new insight into potential genetic and environmental influences on disease biology. These findings highlight the potential for the development of biomarker- and patient-targeted therapeutic strategies in MF.

## 1. Introduction

Mycosis fungoides (MF) is the most common subtype of cutaneous T-cell lymphoma (CTCL), accounting for approximately 50% of cases [1]. MF is classified as a primary cutaneous lymphoma arising from skin-homing CD4⁺ T cells, characterized histopathologically by epidermotropism of atypical helper T lymphocytes [2,3,4]. MF progresses in stages from erythematous patches to plaques and tumors, with advanced stages spreading to lymph nodes, blood, and visceral organs, significantly decreasing survival [3,5].

MF is a rare malignancy with an estimated annual incidence of 6.4 cases per million people [6]. It primarily affects individuals over age 50 and is more common in men, with a male-to-female ratio of approximately 2:1 [7,8]. While more common in African Americans and Hispanics, MF remains most prevalent among Caucasians due to demographic size [9,10]. Risk factors include chronic smoking, obesity, eczema, and occupational exposures such as farming, woodworking, and painting [10,11].

Diagnosing MF remains a clinical challenge, particularly in early stages when it mimics benign inflammatory dermatoses. Skin biopsy is the gold standard [3] and is supported by ancillary tests such as T-cell receptor gene rearrangement, flow cytometry, and immunohistochemical markers including TOX, CCR4, and CLA [12,13]. Disease staging follows the TNMB system, which is crucial in guiding treatment decisions [14]. Early-stage MF (IA–IIA) is typically managed with skin-directed therapies like topical corticosteroids, phototherapy, and localized radiation, while systemic agents, retinoids, interferon-α, HDAC inhibitors, and monoclonal antibodies, are reserved for advanced stages (IIB–IV) [15,16]. Despite available treatments, prognosis remains poor, with a 10-year survival of 42% in tumor-stage MF and a 5-year survival of 27% in advanced CTCL [5,17].

Over the past decade, genomic studies have identified recurrent alterations in tumor suppressor genes (*TP53*, *FAT1*, *KMT2D*, *TET2*) and signaling molecules (*JAK3*, *STAT3*) [5,17,18]. Epigenetic dysregulation also plays a key role in MF, with frequent mutations in chromatin modifiers (*SETD2*, *DNMT3A*) and methylation regulators (*TET2*, *SOCS1*) [1,13]. These findings implicate disruptions in DNA repair, apoptosis, and cytokine signaling in MF pathogenesis. However, small sample sizes, inconsistent methodologies, limited cohort diversity [5,17,18], low tumor cell content in lesional skin, and diagnostic overlap with Sézary syndrome [19] hinder past efforts, leaving MF’s genomic landscape incomplete and underscoring the need for deeper molecular profiling.

This study seeks to address the under-characterization of MF by evaluating its mutational landscape using a large, diverse cohort derived from a publicly accessible genomic repository. By identifying recurrent mutations and disrupted pathways, our findings aim to expand the molecular framework of MF to improve diagnostic precision, risk stratification, and guide more effective treatment strategies.

## 2. Materials and Methods

This study was deemed exempt from institutional review board oversight by Creighton University (Phoenix, AZ, USA) because it involved analysis of de-identified, publicly available data from the AACR Project GENIE database. Clinical and genomic information from 2017 onward was accessed through the cBioPortal platform (version 17.0-public) on 5 June 2025. The AACR GENIE database compiles genomic sequencing data from 19 international cancer centers, reflecting a range of sequencing methodologies. Genomic sequencing in the AACR GENIE database is conducted using unbiased whole-genome/exome sequencing or targeted panels with up to 555 genes. In this dataset, all samples were sequenced using targeted next-generation sequencing (NGS) panels, with the majority processed using DFCI-ONCOPANEL-3 (32%), MSK-IMPACT-HEME-468 (20%), and MSK-IMPACT-HEME-400 (14%). No samples in this cohort were sequenced using whole-exome sequencing (WES) or whole-genome sequencing (WGS). Sequencing depth typically exceeded 500×, consistent with expectations for targeted panels. Regarding sample composition, approximately 65% of specimens were derived from primary tumors and 100% of cases were tumor-only samples. Matched tumor-normal information was not explicitly annotated, limiting the ability to exclude germline variants across the cohort.

Although each participating institution uses its own internal pipeline for mutation calling and annotation, all data were standardized according to GENIE harmonization protocols via Genome NEXUS. This includes commonly used tools such as GATK for variant detection and ANNOVAR for annotation, though specific software and versions vary by institution. It is important to note that differences in bioinformatic pipelines may exist both across and within contributing institutions. While therapeutic response and clinical outcome data are available for certain cancer types in the database, treatment details were not documented for MF.

Our patient cohort included individuals diagnosed with MF, selected from a broader dataset of mature T-cell and natural killer (NK) cell neoplasms. Tumor samples were categorized as either primary (from the original tumor location) or metastatic (from secondary sites). To assess differences in alteration frequencies per gene between primary and metastatic groups, a chi-squared test was applied according to the prevalence of mutations in each group. The dataset contained genetic information, histologic classification, and patient demographics, including sex, age, and race. Although targeted sequencing panels varied across institutions, most captured commonly mutated cancer-related genes like *FAT1*, *KMT2D*, and *TP53*. Genes without established clinical relevance were typically excluded from panel designs, and mutations classified as synonymous were omitted. Structural variants and copy number alterations (CNAs) were not analyzed. Patients with incomplete data were excluded from this study as well. Accounting for factors like panel size helps mitigate platform variability and improves comparability across different sequencing assays.

All analyses were carried out using R/R Studio (R Foundation for Statistical Computing, Boston, MA, USA), with a *p*-value < 0.05 considered statistically significant. For continuous variables, results are expressed as mean values ± standard deviations (SD). Categorical data are described by absolute counts and percentages. The chi-squared test was used to explore associations between categorical variables. To compare continuous variables between two independent groups, data distribution was first assessed. Normally distributed variables were compared using a two-sided Student’s *t*-test, while non-normally distributed variables were analyzed with the Mann–Whitney U test. To account for the possibility of false positives arising from multiple hypothesis testing, adjustments were applied using the Benjamini–Hochberg false discovery rate (FDR) correction (q < 0.05) for all relevant analyses, including the racial enrichment and co-occurrence tests. Confidence intervals for co-occurrence proportions were calculated using the Clopper–Pearson (exact) method for binomial data, which provides conservative but widely accepted estimates, particularly appropriate for small sample sizes.

Mutation data were obtained from the AACR GENIE harmonized MAF (mutation annotation format) files, which ensure consistent variant annotations, such as standardized gene names and protein changes, across institutions. For this analysis, only nonsynonymous alterations, including missense, truncating, in frame, and splice-site mutations, were retained. Variants were further filtered based on a minimum variant allele frequency (VAF) threshold of 5% and sequencing depth of at least 100×.

## 3. Results

### 3.1. Mycosis Fungoides Patient Demographics

Given the relatively small number of MF cases in available genomic datasets, adult, pediatric, primary, and metastatic tumor specimens were grouped together for all analyses. Details regarding patient demographics can be found in Table 1. A total of 156 tumor samples from 147 individuals were considered. Of these patients, 83 (56.5%) were male and 61 (41.5%) were female. Analysis of ethnicity revealed that 129 individuals (87.8%) were non-Hispanic/non-Spanish, 7 (4.8%) were Hispanic or Spanish, and ethnicity was not reported for 11 (7.5%) patients. Racial categorization showed that 111 (75.5%) patients were White, 16 (10.9%) were Black, 9 (6.1%) were Asian, 3 (2.0%) identified with other racial backgrounds, and 7 (4.8%) had unreported racial data. Among the samples, 102 (65.4%) were derived from primary lesions, 11 (7.1%) from metastatic sites, and 31 (19.9%) lacked site annotation.

### 3.2. Recurrent Somatic Mutations in MF

**Figure 1:**
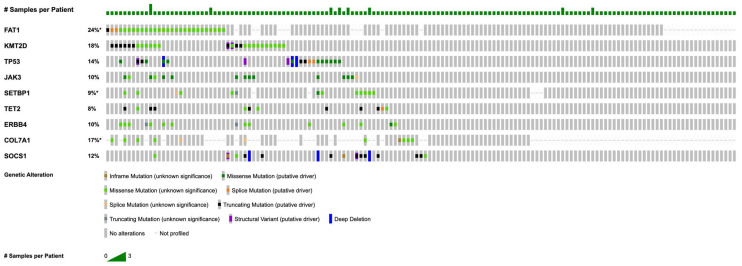
OncoPrint of recurrent mutations in mycosis fungoides (for genes with n ≥ 5, coverage ≥ 100×, VAF ≥ 5%). Asterisk (*) denotes incomplete sample profiling for *FAT1*, *SETBP1*, and *COL7A1*. Note that percentages in the OncoPrint may differ slightly from those in Table 2, as the figure displays only samples profiled for all listed genes to create a complete visual matrix.

Figure 1 illustrates the most commonly detected somatic alterations within this MF cohort, with detailed frequencies provided in Table 2. The most prevalent mutations were in *FAT1* (n = 44; 28.2%), followed by recurrent alterations in *KMT2D* (n = 30; 19.2%), *TP53* (n = 21; 13.5%), *JAK3* (n = 18; 11.5%), *SETBP1* (n = 18; 11.5%), *TET2* (n = 17; 10.9%), *ERBB4* (n = 17; 10.9%), *COL7A1* (n = 17; 10.9%), and *SOCS1* (n = 14; 9.0%).

### 3.3. Mutation Profiles of Frequently Altered Genes

**Figure 2:**
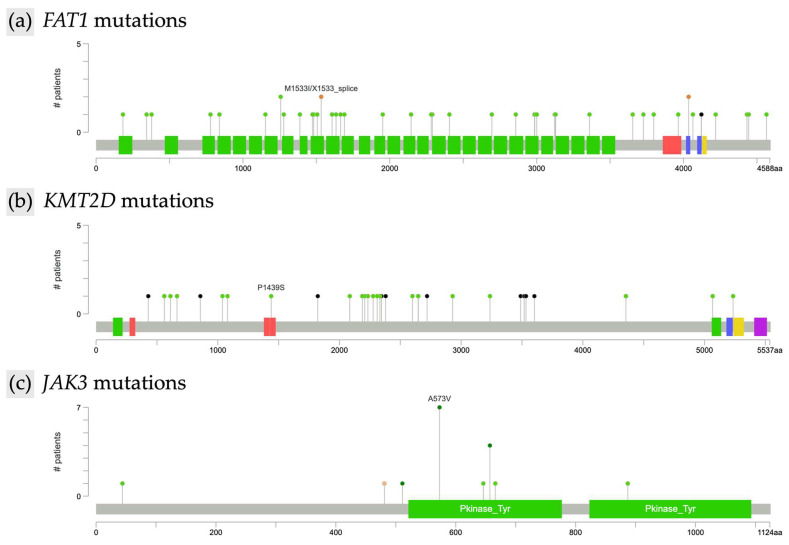
Lollipop plot of recurrent mutations in mycosis fungoides. (**a**) *FAT1*, (**b**) *KMT2D*, and (**c**) *JAK3*.

Further analysis characterized the types of mutations within the most frequently altered genes. In *FAT1* (n = 44), 41 mutations were missense and three were of other types, all resulting in unique protein changes. *FAT1* alterations were scattered across the gene without evidence of recurrent hotspots, as illustrated by the lollipop plot (Figure 2a). In *KMT2D* (n = 30), identified mutations included 14 missense and 6 nonsense alterations, with all *KMT2D* mutations leading to unique protein changes. As with *FAT1*, *KMT2D* mutations were diffusely distributed along the gene and lacked focal clustering (Figure 2b). *TP53* mutations (n = 21) included 15 missense alterations, and all *TP53* mutations were unique at the protein level. *JAK3* mutations (n = 18) were predominantly missense (17 missense, 1 other type). Notably, recurrent hotspot mutations were identified in *JAK3*, including eight instances of the A573V alteration and four distinct changes affecting amino acid position R657 (R657Q, R657W, R657G) (Figure 2c). Among *SETBP1* mutations (n = 18), 16 were missense; all *SETBP1* mutations resulted in unique protein changes. *TET2* alterations (n = 17) included 7 missense and 6 nonsense mutations, and all *TET2* mutations led to unique protein changes. *ERBB4* mutations (n = 17) were primarily missense (n = 15), with two nonsense mutations also identified; all *ERBB4* alterations resulted in unique protein changes. Finally, of the *COL7A1* mutations (n = 17), 13 were missense, and all *COL7A1* mutations were unique at the protein level.

### 3.4. Genetic Differences by Sex and Race

When evaluated by sex, no statistically significant gene enrichments were identified after false discovery rate (FDR) correction. However, race-specific enrichments were observed following FDR correction (q < 0.05). Among Asian patients, alterations in *ATP8B1*, *SHH*, *SYNE1*, *OGG1*, *MDM4*, *SPINK4*, and *XPO1* were found exclusively within this group and were significantly enriched (each n = 1; *p* < 0.001). *PRPF40B* mutations were detected in both Asian and White patients but were significantly enriched in Asian patients (n = 1; *p* < 0.001). In Black patients, *EME1* alterations were observed exclusively and showed significant enrichment (n = 1; *p* < 0.001), whereas *ADGRA2* mutations were significantly enriched and occurred only in White patients (n = 1; *p* < 0.001). Racial variations in mutation profiles are summarized in Table 3.

### 3.5. Co-Occurrence and Mutual Exclusivity of Mutations

Among frequently altered genes, notable co-mutation trends emerged. *JAK3* mutations significantly co-occurred with *ERBB4* (n = 10/21; *p* < 0.001), *KMT2D* (n = 9/34; *p* < 0.001), *SOCS1* (n = 7/25; *p* < 0.001), *FAT1* (n = 7/34; *p* = 0.004), and *TET2* (n = 6/22; *p* < 0.001). *KMT2D* alterations were significantly associated with mutations in *FAT1* (n = 13/39; *p* < 0.001), *ERBB4* (n = 9/17; *p* < 0.001), and *TET2* (n = 8/33; *p* < 0.001). *ERBB4* mutations also showed frequent co-occurrence with *FAT1* (n = 8/36; *p* = 0.005) and *TET2* (n = 6/23; *p* < 0.001). Lastly, *SOCS1* alterations showed co-occurrence with *SETBP1* (n = 6/24; *p* < 0.001) and *TET2* (n = 5/25; *p* = 0.007). These findings are detailed in Table 4. No gene pairs demonstrated statistically significant mutual exclusivity (*p* > 0.100 for all comparisons).

## 4. Discussion

### 4.1. Genomic Landscape and Demographic Trends

Utilizing the AACR Project GENIE dataset, this study defines the mutational profile of mycosis fungoides (MF). Comprehensive analysis identified the most frequently mutated genes and revealed significant co-occurrence patterns, along with race-specific enrichments of low-frequency alterations. These findings provide new insights into the molecular complexity of MF and highlight the role of disrupted signaling, epigenetic regulation, and immune pathways in its pathogenesis.

Our cohort was predominantly male (56.5%) and White (75.5%), consistent with established MF demographics, which report a higher incidence in middle-aged men, with Caucasians being the most frequently affected group due to their larger representation in the general population [6,8,10]. Following FDR correction, no statistically significant sex-based mutational differences were observed; however, several race-specific mutations emerged. Asian patients exhibited enrichment of *ATP8B1*, *SHH*, *SYNE1*, *OGG1*, *MDM4*, *SPINK4*, *XPO1*, and *PRPF40B*, whereas *EME1* was significantly enriched in Black patients, and *ADGRA2* in White patients. These novel associations suggest underlying genetic or environmental influences that merit further exploration, especially given the disproportionate burden of early-onset and more aggressive disease among African American and Hispanic populations [6,8,9,10]. Nonetheless, these race-specific enrichments must be interpreted with caution. The statistical power of this sub-analysis is severely limited by the small number of patients in the Asian (n = 9) and Black (n = 16) cohorts, making the results susceptible to statistical instability. These associations, while statistically significant after FDR correction, are based on single-event mutations and require validation in larger, more diverse cohorts before any definitive conclusions can be drawn.

### 4.2. Commonly Mutated Genes and Pathways

Considerable genetic heterogeneity was exhibited by our MF cohort, with recurrent mutations observed in genes regulating cell adhesion, epigenetic modification, cytokine signaling, and DNA repair. The most frequently mutated genes were *FAT1* (28.2%), *KMT2D* (19.2%), *TP53* (13.5%), *JAK3* (11.5%), *SETBP1* (11.5%), *TET2* (10.9%), *ERBB4* (10.9%), *COL7A1* (10.9%), and *SOCS1* (9.0%). These findings align with prior studies reporting *FAT1*, *KMT2D*, *TP53*, *JAK3*, *TET2*, and *SOCS1* as key contributors to MF biology [1,5,13,17,18]. Notably, *ERBB4* and *COL7A1* are not emphasized in MF literature despite their relatively high prevalence in our cohort and may warrant further exploration as underrecognized contributors to MF.

The most frequently mutated genes in our dataset affect a range of biological processes. *TP53* is a central regulator of DNA damage response and apoptosis, while *KMT2D*, *TET2*, and *SETBP1* are epigenetic modifiers that control chromatin structure and transcription [5,20]. *JAK3* and *SOCS1* are key components of the JAK/STAT cytokine signaling pathway, regulating cell proliferation and survival [13,17]. *FAT1* modulates Wnt signaling and cell adhesion, whereas *ERBB4* activates receptor tyrosine kinase pathways that promote cell growth [21,22]. Although the role of *COL7A1* in MF remains unclear, it plays an essential role in dermal-epidermal junction integrity by encoding collagen VII. This suggests its alteration may reflect broader changes in skin-specific extracellular matrix biology [23].

While *JAK3* demonstrated a focal hotspot at A573V, most mutations, including those in *FAT1* and *KMT2D*, were widely dispersed across their coding regions without evidence of consistent clustering. This diffuse distribution pattern is characteristic of tumor suppressor and chromatin-modifying genes, where loss-of-function arises from diverse disruptive variants rather than single-site activating mutations. The absence of clearly defined driver events in *FAT1* and *KMT2D* aligns with the broader genomic heterogeneity observed in mycosis fungoides, which has been described as lacking a unifying mutational signature [5,19]. Nevertheless, the recurrent involvement of pathways, including p53, epigenetic modifiers, JAK signaling, and Wnt signaling, supports the development of targeted therapeutics.

It is important to recognize that our findings should be interpreted in the context of the broader MF genomic literature, which reveals some variability. For instance, a recent comprehensive study by Fléchon et al. using WES identified recurrent *JUNB* alterations (13%) as a key driver in MF, a finding not prominent in our cohort. Conversely, our study identified *FAT1* as the most frequently mutated gene (28.2%), an alteration not emphasized in their analysis. These discrepancies likely stem from methodological differences: the targeted sequencing panels used in the GENIE database may not be designed to capture the complex structural variants or non-coding mutations affecting *JUNB*, while the higher prevalence of *FAT1* in our cohort may reflect the specific gene inclusion on these panels or differences in cohort characteristics [24].

### 4.3. p53 Pathway

In our cohort, *TP53* mutations were present in 13.5% of cases, aligning with previous literature reporting this mutation in 24% of tumor-stage MF cases [5]. p53 is a tumor suppressor that plays a critical role in genomic surveillance by triggering cell cycle arrest or apoptosis in response to DNA damage, preventing the survival of genetically damaged cells. Loss of *TP53* function disrupts G1/S checkpoint control, allowing abnormal cells to evade apoptosis, continue dividing, and accumulate additional mutations. These findings support a model in which *TP53* inactivation contributes to genomic instability and the proliferation of pathogenic lymphocytes classically seen in MF [25].

This alteration has been associated with advanced disease, poor prognosis, and chemotherapy resistance in MF and related CTCLs, underscoring its significance in disease progression [5,20]. Its presence, therefore, indicates a more aggressive disease phenotype and may influence the consideration of more intensive therapeutic strategies. Additionally, *TP53* mutations may help guide treatment selection towards therapies that induce apoptosis of neoplastic T cells through p53-independent pathways, such as psoralen plus ultraviolet A (PUVA) therapy, which is commonly used in the treatment of early-stage disease and remission maintenance [26]. 

### 4.4. Epigenetic Modification

Epigenetic modification is the regulation of gene expression through DNA methylation and chromatin remodeling. Dysregulation of these processes has been recognized as a hallmark of MF [1]. A recent study reported mutations in epigenetic regulators in 45.78% of MF cases, with 37.85% involving genes related to DNA methylation [20]. Similarly, our cohort demonstrated frequent mutations in regulators, including *KMT2D* (19.2%), *TET2* (10.9%), and *SETBP1* (11.5%).

*KMT2D* encodes a histone methyltransferase that facilitates H3K4 methylation and gene activation. Loss-of-function mutations in *KMT2D* are frequently reported in MF and lead to altered gene expression and tumor suppressor loss. These mutations are associated with poor prognosis in peripheral T-cell lymphomas (PTCLs), with evidence suggesting *KMT2D* mutations independently predict worse overall survival [1,17,20]. Therapeutically targeting *KMT2D* holds promise, as emerging preclinical data suggests KDM5 inhibitors may restore H3K4 methylation and suppress tumor growth in *KMT2D*-mutant lymphomas [27].

*TET2* contributes to DNA demethylation and is essential for regulating gene expression and immune cell differentiation. Mutations lead to global DNA hypermethylation, altered gene expression, and contribute to lymphomagenesis and disease progression [28]. In the broader context of T-cell lymphomas, *TET2* mutations were identified in 53% of PTCLs and were associated with adverse clinical features, including advanced stage and poor prognosis [20]. 

*SETBP1* mutations have yet to be discussed in the context of MF. However, studies on gain-of-function mutations in myeloid malignancies describe its role in promoting transcriptional dysregulation by upregulating genes associated with development and proliferation, enhancing oncogenic gene expression [29]. Together, these epigenetic alterations reflect an ongoing loss of transcriptional regulation and contribute to the immune dysregulation and cellular plasticity observed in MF.

### 4.5. JAK/STAT Signaling

Aberrant activation of the JAK/STAT signaling axis has emerged as a central mechanism in MF pathogenesis. In our analysis, *JAK3* mutations occurred in 11.5% of samples with a hotspot alteration of A573V, a point mutation previously reported in MF with a variant allele frequency of 41.67%. This specific point mutation has been previously shown to be a gain-of-function alteration that activates IL-2-mediated signaling, promoting malignant T-cell proliferation and survival, thus representing a key oncogenic driver in a subset of MF patients [5]. Our identification of *JAK3* mutations is consistent with its established role in MF pathogenesis; however, reported frequencies vary across studies, with 8.3% in MF tumor-stage samples in McGirt et al. and 33.3% in MF patients in Bastidas Torres et al., underscoring the influence of sequencing platforms and cohort selection on the reported genomic landscape [5,17]. 

Mutations in *SOCS1* (9.0%), a negative feedback regulator of JAK signaling, further support dysregulation of this pathway in MF. Previous studies have shown that co-alterations of *JAK3* and *SOCS1* amplify downstream STAT signaling and enhance tumor cell survival [13,17]. The presence of both activating mutations and loss of tumor suppressors points to a convergent mechanism of sustained cytokine signaling in MF.

Targeting the JAK/STAT pathway has emerged as a promising therapeutic strategy in MF. Cerdulatinib, a reversible ATP-competitive inhibitor of *JAK1*, *JAK2*, and *JAK3*, demonstrated an overall response rate (ORR) of 35% in a Phase II clinical trial for PTCL [NCT01994382]. Notably, MF patients exhibited the highest response, with an ORR of 45% and complete response of 9%. These findings support cerdulatinib as a well-tolerated and effective treatment for MF, particularly in cases harboring *JAK3* or *SOCS1* alterations [30,31]. Similarly, McGirt et al. demonstrated that three CTCL cell lines were sensitive to the *JAK3* inhibitor tofacitinib. Hut-78, harboring an activating *JAK3* mutation, was most responsive, though HH and MyLa cells also showed sensitivity to *JAK3* inhibition, indicating pathway dependence for growth and survival [5]. In our cohort, activating *JAK3* mutations were found in 11.5% of patients, reinforcing the importance of genomic screening to identify MF patients who may benefit from JAK inhibitors as a targeted, mechanism-based treatment strategy.

### 4.6. Wnt Signaling 

*FAT1* was the most commonly mutated gene in our cohort (28.2%), highlighting its important role in MF pathogenesis. This finding aligns with previous studies reporting *FAT1* alterations in 39% of MF and PTCL cases, where they have been associated with more aggressive disease and poorer overall survival. Loss-of-function mutations in *FAT1* result in aberrant Wnt activation, increased proliferation, and tumor progression. Furthermore, these alterations may compromise epithelial integrity, enhancing cellular motility and invasiveness in MF [18].

### 4.7. Co-Occurrence and Mutual Exclusivity Patterns 

Our study identified significant co-occurrence among several genes in MF, including *JAK3*, *KMT2D*, *ERBB4*, *FAT1*, *TET2*, *SOCS1*, and *SETBP1*, with no evidence of mutually exclusive gene pairs. These co-mutational patterns suggest that MF pathogenesis involves the cooperative disruption of multiple oncogenic pathways rather than isolated single-gene effects. 

Prior studies have reported that *JAK3* mutations often appear alongside alterations in epigenetic regulators such as *KMT2D* and *TET2*, supporting a model of synergistic interactions between cytokine signaling and chromatin remodeling [5]. Additionally, experimental data indicate that concurrent *JAK3* activation and *SOCS1* loss can amplify downstream STAT signaling, reinforcing the biological plausibility of this co-mutational axis in promoting T-cell proliferation and survival [13]. Separate reports have also emphasized the convergence of alterations affecting histone-modifying enzymes, transcription factors, and signaling molecules, though formal co-occurrence testing was not conducted [17]. Together, these observations are consistent with our findings and suggest that genetic cooperation, particularly between immune signaling and epigenetic dysfunction, is a fundamental feature of MF.

### 4.8. Limitations

This study has several limitations that should be considered when interpreting the findings. First, the cohort size for MF, although relatively large for a rare cancer, remains limited in statistical power for certain subgroup analyses, particularly those stratified by race or histologic variant. As a result, some findings should be interpreted cautiously and validated in larger, demographically diverse datasets. Second, the cross-sectional nature of the dataset and absence of longitudinal sampling prevent assessment of how mutations evolve during disease progression. This limits our ability to distinguish early driver events from later-occurring passenger mutations. Third, inconsistencies in assay sensitivity, reporting, or gene coverage across platforms could introduce technical bias in mutation frequency estimates. The GENIE consortium aggregates data from multiple centers using different sequencing platforms and pipelines. This heterogeneity means that not all genes were assessed with the same depth or consistency across the entire cohort. For example, a gene included on a large panel at one institution may be absent from a smaller panel at another, potentially leading to an underestimation of its true mutation frequency in the overall cohort. While the GENIE harmonization process mitigates some of these issues, residual batch effects and platform-specific biases cannot be fully excluded. Fourth, the AACR Project GENIE database lacks transcriptomic data, which limits the ability to assess gene expression changes and downstream pathway activation. This is particularly relevant in MF, where overexpression of genes can occur even in the absence of mutations. The lack of transcriptomic and microRNA data also prevents investigation into non-mutational mechanisms of dysregulation, such as altered microRNA expression, which may serve as useful diagnostic or prognostic markers. Fifth, detailed clinical data such as disease stage, risk stratification scores, overall or progression-free survival are not available in the public GENIE dataset for this cohort, precluding any assessment of the prognostic significance of the observed mutations. Sixth, the absence of treatment data in GENIE restricts the ability to evaluate how mutational profiles relate to therapy response or resistance. Without information on therapies received, histologic subtype, or clinical course, we cannot assess how specific genetic alterations may influence treatment outcomes or confound genomic comparisons between primary and relapsed disease. Seventh, while GENIE attempts to exclude redundant samples, the inclusion of multiple tumor specimens from the same patient cannot be entirely ruled out, potentially skewing mutation frequencies. Eighth, all MF cases are analyzed as a single group, without subclassification by disease stage or variant. This aggregation limits the capacity to explore potential subtype-specific mutation patterns or their clinical implications. Ninth, methylation data is not included, preventing analysis of how DNA methylation impacts gene regulation, tumor progression, or treatment response. Tenth, a major limitation of this study is the use of tumor-only sequencing data, which precludes the definitive exclusion of germline variants. Although we applied standard filtering criteria and the GENIE data harmonization pipeline includes filters for common polymorphisms, rare pathogenic germline variants may still be present in the dataset. This could potentially lead to an overestimation of the frequency of certain somatic mutations. Consequently, some of the identified alterations, particularly those with a high variant allele frequency approaching 50% or 100%, could be of germline origin. Therefore, these findings, especially for genes known to harbor pathogenic germline variants, should be interpreted with caution until validated with matched tumor-normal sequencing.

Despite these limitations, this analysis contributes meaningful insights into the molecular profile of MF, highlighting key pathways involved in its pathogenesis and offering potential avenues for future research and therapeutic development.

## 5. Conclusions

This study enhances the current understanding of MF’s mutational profile. Our findings validate the frequent involvement of *FAT1*, *TP53*, *JAK3*, and epigenetic regulators in MF biology, while identifying novel co-mutation and racial enrichment patterns. These findings underscore the need for studies clarifying the impact of these mutations and for clinical trials evaluating pathway-specific therapies and tailored treatment strategies. Future research should prioritize multi-omic integration, longitudinal sampling, and inclusive cohort design to support the development of precision-guided therapies in MF.

## Figures and Tables

**Table 1:** Mycosis fungoides patient demographics (N = 147).

Demographics	Category	N (%)
Sex	Male	83 (56.5)
	Female	61 (41.5)
	Unknown	3 (2.0)
Age Category	Adult	145
	Pediatric	2
Ethnicity	Non-Hispanic	129 (87.8)
	Unknown/Not Collected	11 (7.5)
	Hispanic	7 (4.8)
Race	White	111 (75.5)
	Black	16 (10.9)
	Asian	9 (6.1)
	Other	3 (2.0)
	Unknown	7 (4.8)
Sample Type	Primary	102 (65.4)
	Metastasis	11 (7.1)
	Not Collected	31 (19.9)

**Table 2:** Mutation frequency in mycosis fungoides.

Gene	N (%)
*FAT1*	44 (28.2)
*KMT2D*	30 (19.2)
*TP53*	21 (13.5)
*JAK3*	18 (11.5)
*SETBP1*	18 (11.5)
*TET2*	17 (10.9)
*ERBB4*	17 (10.9)
*COL7A1*	17 (10.9)
*SOCS1*	14 (9.0)

**Table 3:** Somatic mutation enrichment by race.

Gene (Chi-Squared)	Asian, N (%)	Black, N (%)	White, N (%)	*p* Value
*ATP8B1*	1 (100.00)	0 (0.00)	0 (0.00)	*p* < 0.001
*SHH*	1 (50.00)	0 (0.0)	0 (0.00)	*p* < 0.001
*SYNE1*	1 (50.00)	0 (0.0)	0 (0.00)	*p* < 0.001
*OGG1*	1 (33.33)	0 (0.0)	0 (0.00)	*p* < 0.001
*ADGRA2*	0 (0.00)	0 (0.0)	1 (100.00)	*p* < 0.001
*EME1*	0 (0.00)	1 (25.00)	0 (0.00)	*p* < 0.001
*MDM4*	1 (11.11)	0 (0.00)	0 (0.00)	*p* < 0.001
*SPINK4*	1 (11.11)	0 (0.00)	0 (0.00)	*p* < 0.001
*XPO1*	1 (11.11)	0 (0.00)	0 (0.00)	*p* < 0.001
*PRPF40B*	1 (100.00)	0 (0.00)	1 (3.70)	*p* < 0.001

**Table 4:** Significant co-occurring mutations in mycosis fungoides.

A	B	N (%)	95% CI	*p* Value
*JAK3*	*ERBB4*	10 (47.6)	25.7–70.2%	*p* < 0.001
*JAK3*	*KMT2D*	9 (26.5)	12.9–44.4%	*p* < 0.001
*JAK3*	*SOCS1*	7 (28.0)	12.1–49.4%	*p* < 0.001
*JAK3*	*FAT1*	7 (20.6)	8.7–37.9%	*p* = 0.004
*JAK3*	*TET2*	6 (27.3)	10.7–50.2%	*p* < 0.001
*KMT2D*	*FAT1*	13 (33.3)	19.1–50.2%	*p* < 0.001
*KMT2D*	*ERBB4*	9 (25.7)	12.5–43.3%	*p* < 0.001
*KMT2D*	*TET2*	8 (24.2)	11.1–42.3%	*p* < 0.001
*ERBB4*	*FAT1*	8 (22.2)	10.1–39.2%	*p* = 0.005
*ERBB4*	*TET2*	6 (26.1)	10.2–48.4%	*p* < 0.001
*SOCS1*	*SETB1*	6 (25.0)	9.8–46.7%	*p* < 0.001
*SOCS1*	*TET2*	5 (20.0)	6.8–40.7%	*p* = 0.007

Co-occurrence percentages were calculated by dividing the number of patients with mutations in both genes by the total number of patients with a mutation in either gene, defined as the sum of those with mutations in A only, B only, or both. Cases where neither gene was altered were excluded from this calculation.

## Data Availability

The data presented in this study are available from the AACR GENIE Database at https://genie.cbioportal.org/ (accessed on 5 June 2025).

## Abbreviations

The following abbreviations are used in this manuscript:

ATP	Adenosine Triphosphate
*ADGRA2*	A Disintegrin and G Protein–Coupled Receptor A2
AACR	American Association for Cancer Research
ANNOVAR	Annotate Variation
CNAs	Chromosomal Copy Number Alterations
CLA	Circulating Leukocyte Antigen
CD4+	Cluster of Differentiation 4 Positive
CTCL	Cutaneous T-Cell Lymphoma
*DFCI-ONCOPANEL-3*	*Dana-Farber Cancer Institute OncoPanel 3*
DNA	Deoxyribonucleic Acid
*DNMT3A*	DNA Methyltransferase 3 Alpha
*EME1*	*EME1* Homolog, DNA Repair Endonuclease Subunit
*ERBB4*	Epidermal Growth Factor Receptor 4
*XPO1*	Exportin 1
FDR	False Discovery Rate Correction
*FAT1*	FAT Atypical Cadherin 1
GATK	Genome Analysis Toolkit
GENIE	Genomics, Evidence, Neoplasia, Information, Exchange
*NEXUS*	Genome *Nexus*
HDAC inhibitors	Histone Deacetylase Inhibitors
*KDM5*	Histone Lysine Demethylase 5
*IL-2*	Interleukin-2
*JAK1*	Janus Kinase 1
*JAK2*	Janus Kinase 2
*JAK3*	Janus Kinase 3
*KMT2D*	Lysine Methyltransferase 2D
MSK	Memorial Sloan Kettering
*MSK-IMPACT-HEME-400*	Memorial Sloan Kettering Integrated Mutation Profiling of Actionable Cancer Targets Hematologic Panel with 400 genes
with 468 genes	*MSK-IMPACT-HEME-468*
microRNA	MicroRNA
*MDM4*	Mouse Double Minute 4 Homolog
MAF	Mutation Annotation Format
MF	Mycosis Fungoides
NK	Natural Killer
NGS	Next-Generation Sequencing
ORR	Objective Response Rate
*OGG1*	8-Oxoguanine DNA Glycosylase
PTCLs	Peripheral T-Cell Lymphomas
*P53*	*p53* Tumor Suppressor
*PRPF40B*	Pre-mRNA-Processing Factor 40 Homolog B
*SETBP1*	SET Binding Protein 1
*SETD2*	SET Domain Containing 2
*STAT3*	Signal Transducer and Activator of Transcription 3
*SHH*	Sonic Hedgehog Signaling Molecule
*SPINK4*	Spink Serine Peptidase Inhibitor Kazal Type 4
SD	Standard Deviation
*SOCS1*	Suppressor of Cytokine Signaling 1
*SYNE1*	Synaptic Nuclear Envelope Protein
*TOX*	Transcription Factor TOX
*TP53*	Tumor Protein *p53*
VAF	Variant Allele Frequency
WES	Whole-Exome Sequencing
WGS	Whole-Genome Sequencing
Wnt	Wnt Signaling Pathway
